# The combination of physical activity with fruit and vegetable intake associated with life satisfaction among middle-aged and older adults: a 16-year population-based cohort study

**DOI:** 10.1186/s12877-023-04563-0

**Published:** 2024-01-09

**Authors:** Richard Szewei Wang, Yu-Ni Huang, Mark L. Wahlqvist, Thomas T. H. Wan, Tao-Hsin Tung, Bing-Long Wang

**Affiliations:** 1 Tsinghua-Berkeley Shenzhen Institute, Tsinghua University, Shenzhen, 518055 China; 2 Evidence-Based Medicine Center, Taizhou Hospital of Zhejiang Province Affiliated to Wenzhou Medical University, Linhai, 317000 Zhejiang China; 3 College of Medical and Health Science, Asia University, Taichung, 41354 Taiwan; 4 Monash University, Melbourne, 3800 Australia; 5 School of Global Health Management and Informatics, University of Central Florida, Orlando, FL 32816 USA; 6 School of Health Policy and Management, Chinese Academy of Medical Sciences & Peking Union Medical College, Beijing, 100730 China

**Keywords:** Older adults, Life satisfaction, Physical activity, Fruit and vegetable intake, Personal behaviors, Health promotion

## Abstract

**Background:**

Life satisfaction (LS) is part of a positive psychological feeling that protects individuals from a physical decline in old age. A healthy lifestyle, including physical activity (PA) and a healthy diet, such as the intake of fruits and vegetables (F&V), can lead to a better experience of LS in older adults. However, the association between PA and F&V intake habits when occurring together in older adults is still unclear for LS. The study aimed to investigate the combined association of PA and F&V intake on LS among a cohort of older Taiwanese adults.

**Methods:**

Five waves of population-based data gathered by the Taiwan Longitudinal Survey on Aging between 1999 and 2015 were analyzed. The year 1999 was set as the baseline, and the number of respondents was 4,440. The independent variables included the frequency, duration, and intensity of PA and the frequency of F&V intake. LS was assessed by using the Life Satisfaction Index. We performed generalized estimating equations (GEE) analysis with adjustment for covariates of health behaviors and health indicators.

**Results:**

After adjusting for confounders, model 1 showed that moderate and high-PA levels significantly correlated with LS (odds ratio [OR] = 1.41, 95% CI = 1.12–1.79) and OR = 1.74, 95% CI = 1.50–2.02). Moreover, high-F&V intake significantly correlated with LS (OR = 2.07, 95% CI = 1.69–2.53). Regarding the combined association shown in model 2, compared with both the low PA and F&V intake group, there were significantly higher LS in the both-high-group (OR = 4.69, 95% CI = 3.49–6.31), only-high-F&V intake (OR = 2.87, 95% CI = 2.14–3.85), only-high-PA (OR = 2.48, 95% CI = 1.74–3.52).

**Conclusions:**

Our findings show the significant combined association of PA and F&V intake on LS among older adults. In addition, older adults who engaged in higher frequency, duration, and intensity of daily PA combined more than seven times a week of F&V intake had significantly higher LS than those who only engaged in low PA or only intake less F&V. Adopting multiple healthy behaviors in daily life is a safe and effective approach to promote LS among older adults.

## Introduction

Worldwide, there is an increasingly aging population. According to the World Health Organization (WHO), the number of people aged over 65 years will reach two billion by 2050. About 25% of older adults present a mental disorder during their lifetime, which exerts a considerable health burden [1]. Accordingly, there is a need for cross-disciplinary actions comprising the provision of health and evidence-based adoption of life behaviors, including physical activity (PA) and nutritional intake [2–4].

It is generally recognized that positive well-being has three dimensions: positive affect, negative affect, and life satisfaction (LS) [5]. Previous studies have shown an association between happiness and healthier and longer lives [6–9]. The LS indicator determines an individual's positive emotions and assesses how well an individual's expectations of life match their actual state [10]. Measures of life satisfaction are more stable than positive emotions because they reflect subjective feelings of success and happiness [10, 11]. LS protects individuals against the physical decline in old age [12]. In addition, LS was significantly and negatively associated with morbidity in healthy and ill populations [9] and was associated with lower morbidity and mortality in community-dwelling older individuals [8].

There is increasing evidence that primary health-related behaviors, including regular PA and wholesome dietary patterns, may improve the intrinsic abilities of older adults [1]. Healthy life behaviors, including PA and good nutrition, can allow older adults to achieve their goals, feel capable, maintain a sense of identity, and experience improved life satisfaction (LS) [13]. Among older adults, PA is positively correlated with LS [14, 15]. However, while PA is vital for health, most older adults do not meet the PA recommendations [16]. Moreover, fruit and vegetable (F&V) intake is positively correlated with mental and physical health as well as LS [17], especially among older adults [18–20]. Furthermore, F&V intake is negatively correlated with the risk of depressive symptoms and cognitive decline [21, 22]. Research has consistently demonstrated a positive correlation between fruit and vegetable (F&V) intake and mental health especially depression symptoms. For example, vegetables vitamins A, B, C, fibers, plant-based food correlated negatively with depression [23, 24]. These findings collectively support the idea that increased F&V intake may reduce depression risk through various nutritional and neurological mechanisms. However, the long-term and combined association of PA and F&V intake on LS remain unclear. Life satisfaction is particularly important for the mental health of older adults [25]. Therefore, physical activity, dietary intake, and life satisfaction are all highly relevant issues to the health of older adults. Thus, we aimed to examine the combined associations of PA and F&V intake on LS among middle-aged and older Taiwanese adults.

## Methods

### Data and Sample

**Fig. 1:**
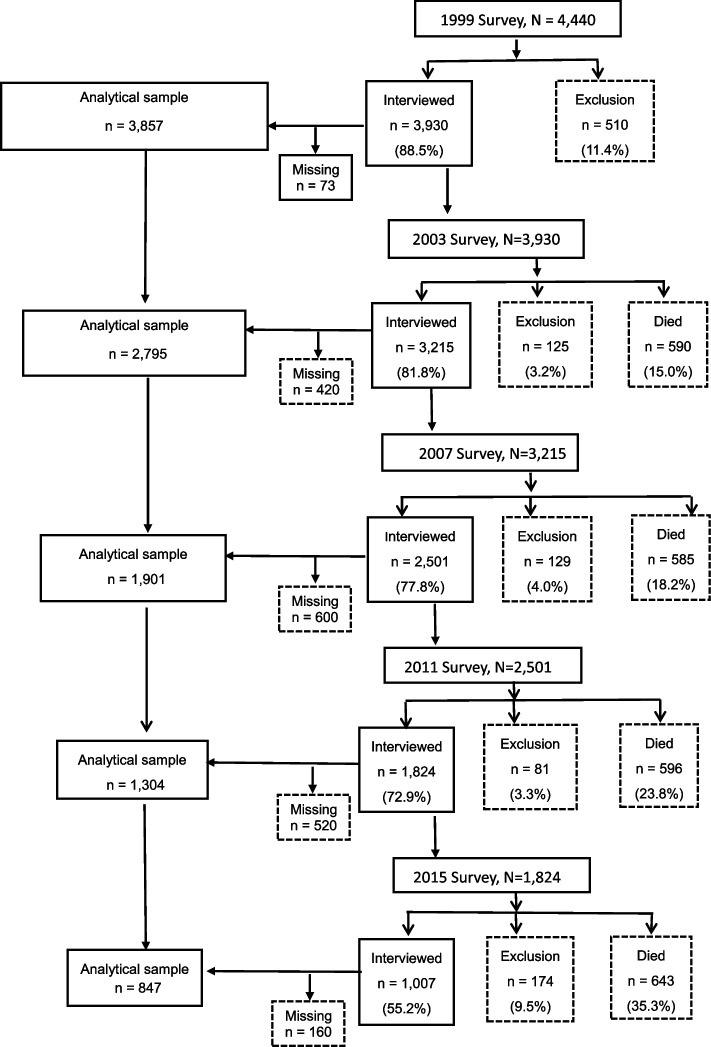
Participants in serial surveys in the TLSA from 1999 to 2015

The data were retrieved from the Taiwan Longitudinal Survey on Aging (TLSA) survey data from 1999 to 2015, a joint initiative of the Taiwan Health Promotion Administration and the Population Studies Center of the University of Michigan [26]. The TLSA represents the Taiwan region with a long-term follow-up period, high completion rate, and relocation case tracking [26]. The first wave of TLSA was conducted in 1989, with waves of surveys conducted every 3–4 years and the sixth wave of surveys completed in 2015. The participants were selected using a stratified, multi-stage, random sampling method. This database comprised data collected from the household-registered population aged over 60 years who were registered in 311 Taiwanese townships and cities, sampled neighbors from 56 townships and urban areas, and selected two adults aged over 60 years from each sample as interview cases. In addition, in 1996 and 2015, a supplementary nationally representative sample of individuals aged 50–56 years was collected using the sampling method applied in the baseline survey [27]. Detailed information about the study design and sampling of TLSA has been presented in the published research articles [28, 29]. The fourth wave of TLSA conducted in 1999 contained data on diet, nutrition, and life satisfaction index (LSI), which were consistent with the objectives of our study. Therefore, we set the 1999 data as a baseline and created categorical variables to differentiate the data using data from 2003, 2007, 2011, and 2015 to analyze the association between PA and F&V intake and LS over 16 years. After excluding the number of missing and deceased during the follow-up period, respondents who completed five waves of interviews and self-reports were included in the analysis. The number of respondents in each wave was: 4,440 (1999), 3,930 (2003), 3,215 (2007), 2,501 (2011), and 1,824 (2015). Exclusion is lost-to-follow-up mainly due to moving and rejection to be interviewed; missing is incomplete data on significant variables (Fig. 1).

### Outcome measure

Life satisfaction (LS) was measured using the Life Satisfaction Index (LSI) [30], which comprises ten items [31] with higher scores indicating higher LS levels. The ten items are: (1) Is your life better than most people's? (2) Are you satisfied with your life? (3) Are you interested in what you do? (4) Have the last few years been the best in your life? (5) If possible, would you want to take another path and start your life over again? (6) Do you expect something happy to happen in the future? (7) Do you think your life should be better than it is now? (8) Do you feel that most of what you do is monotonous and of no interest? (9) Do you feel that you are old and that life is boring? (10) Would you say your life has matched your expectations? as "yes" or "no"? The items were reverse-scored when necessary and summed; accordingly, higher LSI scores correspond with higher LS (Cronbach's α = 0.73–0.81) [31]. The LSI has a score range of 0 to 10; a total score ≥ 6 was considered as feeling satisfied with life and a higher level of LS [31].

### Independent variables

Physical activity (PA) was analyzed as an independent variable. We measured PA by considering the frequency, duration, and intensity of daily physical activity following the methodology of Haase et al. and Pitsavos et al. [32, 33]. Data regarding physical activity were generated from three questions in the questionnaire:

1. "How often do you do routine physical exercise?" and the responses were (a) none (inactive, scored 0), (b) < 2 times/week (scored 1), (c) 3–5 times/week (scored 2) and (d) ≥ 6 times/week (scored 3).
2. If you do physical exercise, "How many minutes do you spend each time?" and the responses were (a) < 15 min/time (scored 1), (b) 15–30 min/time (scored 2), and (c) ≥ 30 min/time (scored 3).
3. "Do you sweat or pant after doing exercise?" and the responses were (a) no sweating or panting (scored 1) and (b) some or lots of sweating or panting (scored 2).

Each participant's total PA score was obtained by multiplying the scores of the three questions, and the total score ranged from 0 for inactive people to 18 for highly active people. Next, we calculated the total PA scores and classified them into three groups: low (inactive, total score = 0), moderate (generally active, total score = 1–7), and high (vigorous, total score = 8–18). The methods used in this study were similar to those used in previous relevant studies and were considered acceptable reliability [32–34].

A validated semiquantitative questionnaire was applied to assess the frequency of F&V intake, which in the present study was categorized as follows: "did not eat" as 0 times, < 1 time per week as 0.5 times, 1–2 times per week as 1.5 times, 3–5 times per week as 4 times, and eating every day as ≥ 6 times a week. The scores for the respective weekly intake of F&V were summed and classified into three groups: low (< 0–6 times), moderate (7–9 times), and high (≥ 10 times).

The combination of PA and F&V intake was classified into five groups: both-low (low PA and low F&V intake), both-high (high PA and high F&V intake), only-high-PA (high PA and low or moderate F&V intake), only-high-F&V intake (high F&V intake and low or moderate PA), and others (low PA and /or moderate F&V intake). Previous publications adopted these selected criteria [21, 22].

### Covariates

Within our generalized estimating equations (GEEs) models, certain covariates such as age and education were treated as time-fixed variables, set at the baseline. Other covariates, such as health statuses and behaviors, were treated as time-varying, allowing them to change over the different waves of the study. Variables included possible related factors of LS: Age divided into three groups of 53–64, 65–74, and > 75; Education adjustment scores were applied for the following groups: ≤ 6, 712, and ≥ 13 years of formal education. Marital status was classified into two categories: yes or no. Regarding marital status, a ‘yes’ was assigned to participants currently married or in a marriage-like relationship, while ‘no’was assigned to participants who were divorced, widowed, separated, or never married. Participants responded yes/no about drinking alcohol: never drink once or twice a week. Participants were classified as nonsmokers or current/past smokers. Tea consumption was classified as < 3 times or ≥ 3 times a week. Hypertension, heart disease, and diabetes were classified based on diagnoses by a physician. Additionally, evidence regarding cardiovascular disease-related diseases was gathered. If the respondent reported having a doctor's diagnosis of heart disease, coronary heart disease, any other heart diseases, stroke, diabetes, or cancer; they were coded as having a history of such diseases.

### Data analysis

All statistical analyses were conducted using IBM SPSS version 22.0. We conducted an analysis using generalized estimating equations (GEEs) with adjustment of time-constant and time-varying covariates [35]. The net influence of PA and F&V intake on LS was examined with the adjustment of covariates. All analyses simultaneously assessed data from five waves of follow-up interviews in 1999 (baseline), 2003, 2007, 2011, and 2015. Data gathered throughout the 16-year study period were assessed simultaneously in all analyses. The baseline measures of LS were included to reduce unobserved heterogeneity. In addition, we took longitudinal models, including LS measurements from previous waves, to examine PA and F&V intake and their associations with LS. All values have been adjusted for weighting according to the study design. In this study, p < 0.05 was considered statistically significant.

## Results

Table 1 shows the baseline characteristics of the participants collected in 1999 regarding PA, F&V intake, and LS. A broader percentage of participants responded to the high PA (men 52.8%, women 45.6%) and F&V intake results (men 79.3%, women 83.7%). Results of the analysis of the combination of PA and F&V intake showed that the group with the highest percentage of males was both high (43.7%), and the group with the highest percentage of females was only F&V intake (39.1%). The results of the analysis of the Life Satisfaction Index (LSI) showed that 70.3% and 64.9% of the male and female participants, respectively, expressed satisfaction (LSI ≥ 6) with their lives.

**Table 1:** Characteristics of participants at baseline of the Taiwan Longitudinal Survey on Aging (1999)

	Total number	Percentage of total number	PA (%)			F &V intake (%)			Combine PA and F&V intake (%)					Satisfied (%)
			Low	Moderate	High	Low	Moderate	High	Both low	Both high	Only high PA	Only high FV	Other	
Sex														
Men	2059	53.4	34.6	12.6	52.8	10.3	10.3	79.3	5.1	43.7	7.2	35.3	8.6	70.3
Women	1798	46.6	44.9	9.5	45.6	7.4	8.9	83.7	3.6	39.1	3.8	44.4	9.1	64.9
Age														
53–64	1401	36.3	45.7	11.6	42.7	8.1	8.8	83.1	4.4	37.4	4.2	45.3	8.6	69.6
65–74	1437	37.3	26.9	9.9	63.2	10.4	9.5	80.1	4.1	50.4	7.9	29.3	8.3	63.2
≧75	1019	26.4	30.6	11.1	58.3	11.1	22.2	66.7	5.6	44.4	11.1	22.2	16.7	66.7
Education														
0–6	2968	77.0	47.3	9.2	43.5	10.6	11.4	78.1	4.9	34.2	6.2	43.5	11.2	62.7
7–12	663	17.2	24.3	13.0	62.7	5.3	5.9	88.8	3.0	58.6	4.7	30.2	3.6	82.8
≧13	226	5.9	10.1	23.2	66.7	2.9	2.9	94.2	2.9	63.8	1.4	30.4	1.4	73.9
Marital status														
Yes	2659	68.9	40.5	10.1	49.4	7.9	9.2	82.9	3.9	42.7	5.0	40.1	8.3	71.1
No	1198	31.1	36.4	15.0	48.6	12.9	11.1	76.0	6.4	36.4	7.5	38.7	11.0	54.3
Smoking														
Yes	954	24.7	47.7	12.8	39.5	17.1	15.9	67.1	11.0	32.6	8.1	33.7	15.1	63.4
No	2903	75.3	37.6	10.7	51.7	6.8	8.0	85.2	2.8	43.7	4.9	41.3	7.3	68.7
Drinking														
Yes	963	25.0	36.7	13.1	50.2	11.5	8.9	79.6	5.9	42.2	7.6	36.7	7.6	70
No	2894	75.0	40.8	10.3	48.9	7.9	9.9	82.3	3.8	41.1	4.8	41.0	9.3	66.7
Hypertension														
Yes	1293	33.5	29.5	14.7	55.8	7.4	10.1	82.5	1.8	45.6	6.9	36.9	8.8	66.4
No	2564	66.5	43.2	9.8	47.0	9.4	9.4	81.2	5.2	40.0	5.1	40.8	8.9	68.1
Diabetes														
Yes	541	14.0	31.4	18.6	50.0	5.7	4.3	90.0	0.0	44.3	1.4	45.7	8.6	70
No	3316	86.8	40.4	10.4	49.2	9.2	10.1	80.7	4.8	41.2	5.9	39.3	8.9	67.4
Heart disease														
Yes	733	19.0	32.5	17.9	49.6	9.5	12.1	78.4	6.0	42.7	5.1	35.0	11.1	59
No	3124	81.0	40.8	10.0	49.2	8.8	9.2	82.0	4.1	41.2	5.6	40.5	8.5	69
Stroke														
Yes	178	4.6	18.8	12.5	68.8	6.7	6.7	86.7	6.3	56.3	6.3	25.0	6.3	50
No	3679	95.4	40.1	11.1	48.9	8.9	9.7	81.4	4.3	41.2	5.5	40.1	8.9	68
Cancer														
Yes	103	2.7	14.3	21.4	64.3	7.1	7.1	85.7	0.0	57.1	0.0	28.6	14.3	71.4
No	3754	97.3	40.1	10.9	49.0	8.9	9.6	81.4	4.4	41.2	5.6	40.0	8.8	67.6
Tea consumption														
≧3	647	17.8	39.7	11.9	48.3	8.0	8.7	83.3	4.0	39.7	6.6	43.0	6.6	68.2
< 3	1258	32.6	38.4	10.1	51.5	6.5	10.1	83.4	3.6	45.9	4.9	37.5	8.1	73.6

Table 2 presents the characteristics of LSI collected from 1999 to 2015. According to the five survey waves data, men are more likely to be satisfied with their lives than women. However, both men and women gradually reduced their LS over time. The percentage of men who felt satisfied with their lives in 1999 was 70.3%, and in 2015 it was 63.8%.

**Table 2:** Characteristics of participants at 1999–2015 of the Life Satisfaction Index

Year		1999		2003		2007		2011		2015		*p*-value
		*N* = 3857		*N* = 2795		*N* = 1901		*N* = 1304		*N* = 847		
Life satisfaction		n	Feel Satisfied (%)	n	Feel Satisfied (%)	n	Feel Satisfied (%)	n	Feel Satisfied (%)	n	Feel Satisfied (%)	
Sex	Men	2059	70.3	1469	65.9	994	64.0	675	64.0	428	63.8	0.1
	Women	1798	64.9	1326	57.3	907	60.4	629	56.8	419	57.5	
Age	53–64	1401	69.6	1181	62.2	942	64.1	768	62.2	569	64.3	< 0.01*
	65–74	1437	63.2	1087	59.5	702	59.1	440	57.4	242	54.5	
	≧75	1019	66.7	527	66.7	257	52.8	96	52.8	36	44.4	
Education	06	2968	62.7	2115	56.7	1406	59.6	933	57.0	609	57.8	0.09
	7–12	663	82.8	505	73.4	360	71.0	269	68.0	169	69.2	
	≧13	226	73.9	175	76.8	135	63.8	102	72.5	69	65.2	
Marital status	Yes	2659	71.1	2054	65.7	1445	64.2	1018	63.4	674	63.2	< 0.01*
	No	1198	54.3	741	45.7	456	54.3	286	49.1	173	50.9	
Smoking	Yes	954	63.4	675	55.8	437	62.2	288	58.7	172	58.1	0.53
	No	2903	68.7	2120	63.1	1464	62.2	1016	60.9	675	61.3	
Drinking	Yes	963	70.0	737	72.6	521	66.7	366	68.4	237	68.4	< 0.01*
	No	2894	66.7	2058	57.4	1380	60.5	938	57.4	610	57.7	
Hypertension	Yes	1293	66.4	927	59.4	586	59.0	371	54.8	217	54.4	< 0.01*
	No	2564	68.1	1868	62.4	1315	63.3	933	62.4	630	62.9	
Diabetes	Yes	541	70.0	362	50.0	203	52.9	121	51.4	70	52.9	0.26
	No	3316	67.4	2433	62.7	1698	63.1	1183	61.3	777	61.4	
Heart disease	Yes	733	59.0	494	52.1	330	53.0	204	51.3	117	48.7	< 0.01*
	No	3124	69.0	2301	63.2	1571	63.7	1100	61.9	730	62.6	
Stroke	Yes	178	50.0	96	75.0	41	75.0	23	43.8	16	50.0	0.09
	No	3679	68.0	2699	61.4	1860	62.0	1281	60.8	831	60.9	
Cancer	Yes	103	71.4	68	64.3	37	42.9	23	50.0	14	50.0	0.84
	No	3754	67.6	2727	61.6	1864	62.5	1281	60.6	833	60.9	
Tea consumption	Yes	647	68.2	488	64.2	352	57.0	227	61.6	235	59.6	0.13
	No	1258	73.6	944	68.4	662	65.1	473	67.4	612	66.1	

^*^P < 0.05

The percentage of women who felt satisfied with their lives in 1999 was 64.9%, and in 2015 it was 57.5%. There was a significant difference in LS among the age groups (*p* < 0.01). Over the study period, the 53–64 years old group achieved an LS rate of 69.6%. Contrastingly, the ≧75 years old group showed the lowest LS rate in 2015 (44.4%), with an annual decrease Moreover, marital status, drinking, hypertension, and heart disease were significantly correlated with LS (Table 2).

**Fig. 2:**
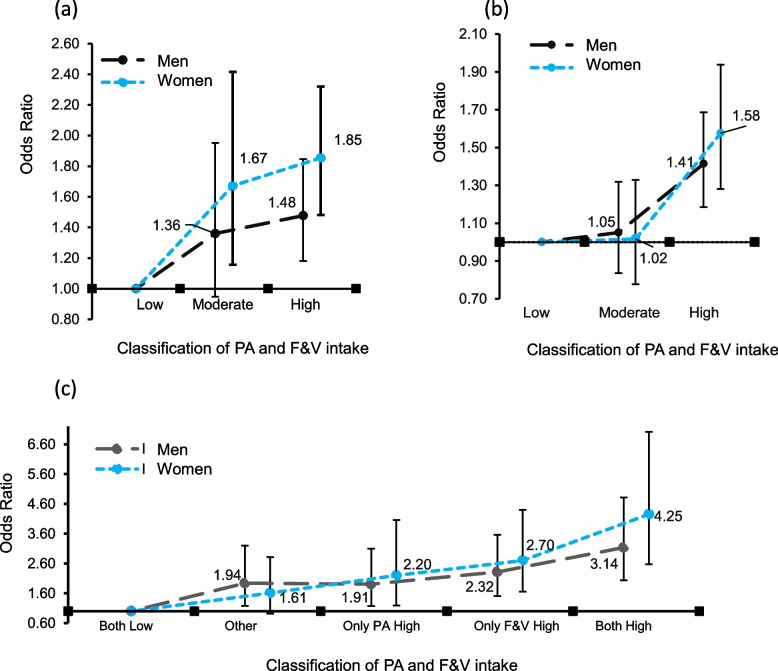
The differences in (**a**)PA, (**b**)F&V intake, (**c**) combined association on LS in sex. These models were adjusted for variables such as health status and behaviors. Bars show the 95% confidence intervals

For Figs. 2, analyses were conducted using specific models to assess the influence of sex and age on the relationship between PA, F&V intake, and LS. These models were adjusted for variables such as health status and behaviors. The adjustments aimed to control for potential confounders, providing a more accurate representation of the relationships. Figure 2 shows the gender differences between PA and F&V intake and the association at LS. As can be observed in Fig. 2a, there was a significant difference in PA for men in the only-high group (odds ratio [OR] = 1.48; 95% CI = 1·16–1·80) and for women in the moderate (OR = 1.67, 95% CI = 0.94–2.40) and high groups (OR = 1.85, 95% CI = 1.4–2.3). Figure 2b shows significant differences in the high-F&V group regardless of gender; the men’s OR was 1.41 (95% CI = 1.15–1.70), and the OR for women was 1.58 (95% CI = 1.20–2.05). Figure 2c also shows that combining PA and F&V had multiple significant differences in LS, and the combined both high group’s OR for men was 3.14 (95% CI = 1.8–4.5), and for women was 4.25 (95% CI = 2.1–6.8).

**Fig. 3:**
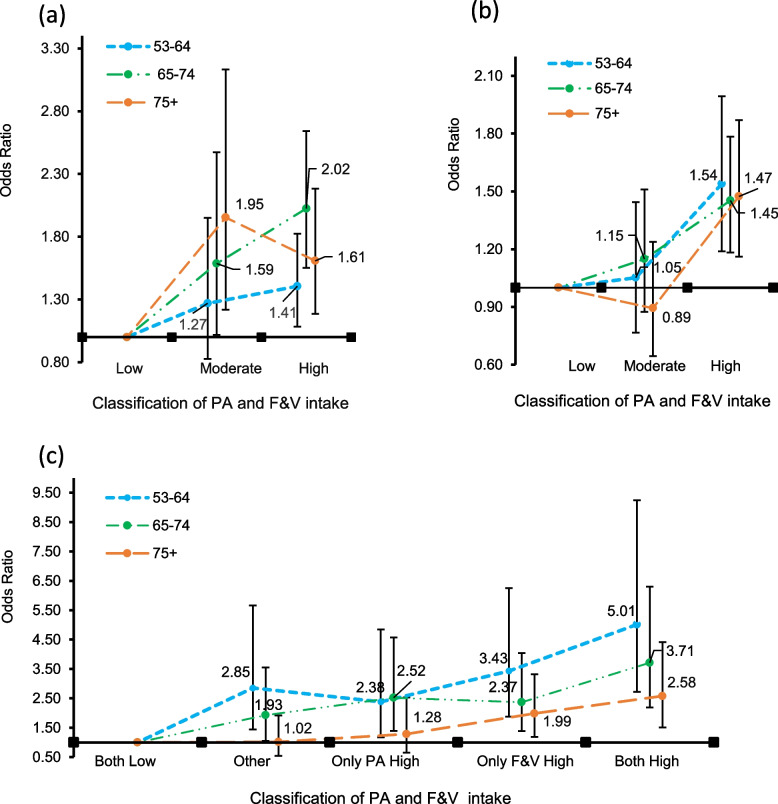
The differences in (**a**)PA, (**b**)F&V intake, (**c**) combined association on LS in age. These models were adjusted for variables such as health status and behaviors. Bars show the 95% confidence intervals

Figure 3 illustrates not only the combined effects of PA and F&V intake on LS but also presents a separate analysis to discern the individual influences of PA and F&V intake on LS across various ages. The three age groups showed significant differences in PA with LS. Only the 53–64 group with moderate PA did not show significant differences (Fig. 3a). The association was more significant in the 65–74 years-old group (OR = 2.02, 95% CI = 1.59–2.73) and ≧75 years-old group (OR = 1.61, 95% CI = 1.05–2.27) than in the 53–64 years-old group (OR = 1.41, 95% CI = 0.94–1.77). Similarly, F&V intake was positively correlated with LS (Fig. 3b). There were multiple significant differences, among which the 53–64 years-old group OR was 1.54;(95% CI = 1.10–2.05), and the 65–74 years-old group (OR = 1.47, 95% CI = 1.10–1.85) and ≧75 years-old groups (OR = 1.45, 95% CI = 1.22–2.78) in high-F&V intake. PA and F&V intake showed a significant combined association on LS except for the ≧75 years-old with an only-PA high group (Fig. 3c). The OR for 53–64 years-old group was 5.01 (95% CI = 2.50–9.50), the OR for 65–74 years-old group was 3.71 (95% CI = 2.22–6.50), and the OR for ≧75 years-old group was 2.58 (95% CI = 1.50–4.30). The result shows that combined PA and F&V intake can significantly promote LS, decreasing age-related association.

We performed GEE regression to analyze the correlation of PA and F&V intake on LS (Table 3). After adjusting for confounders, model 1 showed that moderate and high-PA levels significantly correlated LS (OR = 1.41, 95% CI = 1.12–1.79 and OR = 1.74, 95% CI = 1.50–2.02). Moreover, high-F&V intake significantly correlated with LS (OR = 2.07, 95% CI = 1.69–2.53). Regarding the combined association shown in model 2 (Table 3), compared with the both-low-PA and F&V intake group, there were significantly higher LS levels in the both-high (OR = 4.69, 95% CI = 3.49–6.31), only-high-F&V intake (OR = 2.87, 95% CI = 2.14–3.85), only-high-PA (OR = 2.48, 95% CI = 1.74–3.52), and others groups (OR = 1.87, 95% CI = 1.33–2.64).

**Table 3:** Adjusted longitudinal associations of physical activity and fruit and vegetable intake on life satisfaction

Variables		Life satisfaction	
		**Model 1** **OR (95% CI)**	**Model 2** **OR (95% CI)**
**Independent associations**			
Physical activity	Low	1	
	Moderate	1.41(1.12–1.79)*	
	High	1.74(1.50–2.02)*	
Fruit and vegetable intake	Low	1	
	Moderate	1.19(0.91–1.55)	
	High	2.07(1.69–2.53)*	
Combined physical activity and fruit and vegetable intake	Both low		1
	Both high		4.69(3.49–6.31)*
	Only FV high		2.87(2.14–3.85)*
	Only PA high		2.48(1.74–3.52)*
	Others		1.87(1.33–2.64)*
**Control variables**			
Sex	Men	1	1
	Women	0.95(0.80–1.13)	0.97(0.82–1.15)
Age	53–64	1	1
	65–74	0.83(0.74–1.08)	0.83(0.71–0.98)*
	≥ 75	0.89(0.70–0.98)	0.90(0.74–1.09)
Education	≤ 6	1	1
	7–12	1.62(1.29–2.44)	1.63(1.34–1.98)*
	≥ 13	1.77(1.34–1.98)	1.77(1.29–2.44)*
Marital status	No	1	1
	Yes	1.54 (1.32–1.81)*	1.54(1.31–1.80)*
Smoking	No	1	1
	Yes	0.76(0.63–0.92)*	0.76(0.63–0.92)*
Drinking	No	1	1
	Yes	1.15(0.96–1.38)	1.16(0.96–1.39)
Hypertension	No	1	1
	Yes	0.89(0.76–1.03)	0.88(0.76–1.03)
Diabetes	No	1	1
	Yes	0.95(0.78–1.15)	0.95(0.78–1.16)
Heart disease	No	1	1
	Yes	0.88(0.73–1.05)	0.88(0.73–1.05)
Stroke	No	1	1
	Yes	0.49(0.35–0.69)*	0.48(0.34–0.69)*
Cancer	No	1	1
	Yes	0.61(0.39–0.93)*	0.61(0.40–0.94)*
Tea consumption	< 3	1	1
	≥ 3	1.51(1.30–1.77)*	1.52(1.30–1.77)*

^*^p < 0.05

## Discussion

Our findings demonstrated that PA and F&V intake showed a significant combined association with LS among older Taiwanese adults; moreover, PA and F&V intake each showed a significant positive correlation with LS. Our findings demonstrate the importance of PA and F&V intake in improving LS among older adults in Taiwan.

This study yielded meaningful evidence. First, this was a longitudinal national population-based cohort study on older Taiwanese adults. This is the first 16-year study on the association between PA and F&V intake among older adults in Asia. Second, we demonstrated the combined associations of PA and F&V intake on LS in Taiwanese older adults. Specifically, compared with the both-low PA and F&V intake group, the both-high PA and F&V intake, only-high F&V intake, and only-high PA groups showed a correlation of LS by 469%, 287%, and 248%, respectively. This suggests that concurrently practicing both habits yielded more health benefits than practicing each alone. However, the mechanisms underlying the positive relations of PA and F&V intake on LS remain unclear. The theoretical model of the impact of PA and F&V intake on life satisfaction can be established based on the relationship between quality of life and mental health. The following is a possible theoretical model—a model of how PA and F&V improve life satisfaction. Life satisfaction is a subjective evaluation of a person's quality of life. Quality of life includes factors such as physical, psychological, and social health, as well as meeting basic needs, personal growth, and social interaction [36, 37]. PA helps improve physical health, including enhancing cardiovascular health, maintaining a healthy weight, and enhancing muscle and bone strength. In addition, PA can also release endogenous hormones such as endorphins and dopamine, which can help improve mental health, reduce anxiety and depression, and increase happiness [38–41]. A balanced diet is crucial for maintaining physical health. F&V provides the nutrients the body needs, maintains endocrine balance, enhances immune system function, and prevents chronic diseases. At the same time, F&V intake also related to emotional and mental health, and certain foods can affect neurotransmitter levels, affecting emotions and mood [23, 42]. The impact of action between PA and F&V intake has a positive impact on mental health. Exercise promotes the release of endogenous hormones, enhances self-esteem and confidence, reduces stress, and increases the ability to resist depression and anxiety. A good diet helps stabilize blood sugar levels, reduce emotional fluctuations, and provide the body with the necessary nutrients to support nervous system function [38, 39]. By improving physical and mental health, PA and F&V intake can help improve the quality of life. When individuals feel good physical health, emotional stability, and social satisfaction, they are more likely to feel satisfied with life [40, 41]. Individual differences, cultural differences, social support, and other factors may also affect the impact of PA and F&V intake on life satisfaction. Different people may have different reactions to varying degrees of PA and F&V intake [24, 43]. This model emphasizes the positive impact of PA and F&V intake on life satisfaction, achieved by improving multiple aspects of quality of life. It emphasizes the interactive relationship between physical and mental health, as well as the role of PA and F&V intake in maintaining these health aspects. However, it should be noted that the actual results may vary depending on individual differences and environmental factors. Therefore, it is crucial to develop exercise and dietary plans tailored to the specific circumstances of different individuals to improve life satisfaction. This study has several limitations. First, some of the data were self-reported measures but showed acceptable validity and accuracy. The reliability of the questionnaire interview for assessing food intake is limited; however, it was administered to participants with memory loss. Second, quantitative assessment of food intake is challenging, especially among the older adults; accordingly, we analyzed the intake frequency rather than the quantity (serving size). Third, no reciprocal relationship of the study variables is assumed since the data are limited for performing multi-wave analysis of a panel model. From Fredrickson's broaden–and–build theoretical point of view [44], it is very well possible that feeling better also increases people's activities. Reciprocal causality requires the reconceptualization of the study variables in a panel data study, as noted by Leszczensky & Wolbring [45]. Fourth, due to the limited data, we did not consider the associations of significant SES (such as household income), life events (such as the loss of a partner), and external health system factors (such as policy and economics) on LS. Finally, there also have other residual confounding factors– for examples, there were any neighborhood-level factors that could have considered to adjust for in the analysis (e.g., access to public/private PA facilities, proximity to grocery stores, etc.). Also, other mental health variables (such as anxiety or depression) could have included in the analysis. Future studies should consider personal behaviors and their interplay in LS pathway mediation as antecedents of significant life events. In addition, researchers could facilitate a more in-depth understanding of variations in heart disease risk across different age ranges and may reveal additional correlations and causal relationships by using the autoregressive latent growth curve model [46].

## Conclusion

Our findings confirmed that PA and F&V intake had a significant combined effect on LS among older Taiwanese individuals. These personal actions are safe, effective, and economical ways to associate life satisfaction and promote healthy, safe, and active aging. A standardized and specific program and policy are necessary to promote healthy habits in different older adults, such as daily PA and F&V intake, and one without the other, to improve longevity and healthy aging.

## Data Availability

The datasets used and analyzed during the current study are not publicity available but are available from the https://www.hpa.gov.tw/EngPages/Detail.aspx?nodeid=1077&pid=6197 on request with the permission.
